# Vaginal Bulge

**DOI:** 10.5811/westjem.2015.2.25766

**Published:** 2015-04-06

**Authors:** Kubwimana M. Mhayamaguru, Russel Means, Arthur B. Sanders, Richard Amini

**Affiliations:** * University of Arizona, College of Medicine, Department of Emergency Medicine, Tucson, Arizona; † University of Arizona, College of Medicine, Tucson, Arizona

## PATIENT PRESENTATION

A 61-year-old female presented to the emergency department complaining of constipation and vaginal bulge with valsalva 89 days after a robotic-assisted hysterectomy. The patient had intercourse three days prior to presentation and experienced postcoital abdominal discomfort with vaginal bleeding. She denied any other trauma. She had no other complaints and denies fevers, chills, nausea, vomiting, abdominal distension, or constipation. Physical exam revealed exposed bowel protruding through the vaginal cavity.

## DISCUSSION

This patient had an impressive amount of evisceration through the dehisced vaginal cuff (Figure). Vaginal cuff dehiscence is a rare but emergent complication of gynecologic operations. A full thickness dehiscence can be complicated by prolapse of intra-abdominal organs. When this occurs, evisceration of the distal ileum is most common and can include the appendix as in this case.^2^

Multiple large retrospective studies have demonstrated an increased incidence of dehiscence with laparoscopic hysterectomies (0.64–5.42%) as compared to vaginal hysterectomies (0.13–1.68%).^2^,^3^ This increased risk is likely due to suture knot strength and reduced surgical field visualization.^1^,^2^ Nonsurgical risk factors for dehiscence include post-operative infection, post-menopausal status, exposure to pelvic radiation, corticosteroid use, penetrative vaginal trauma, previous history of vaginal surgery, and coitus prior to full healing of the cuff.^1^ Dehiscence after hysterectomy is most common in the first three months but has been reported as late as five years.^2^,^4^

Vaginal eviscerations are gynecologic emergencies requiring exploratory laparotomy for repair. Prolapsed structures should be irrigated with warm normal saline and wrapped in a moist towel. If delay is anticipated, management includes reduction of prolapsed organs followed by vaginal packing. Because bowel wall edema, peritonitis, and sepsis may result from vaginal dehiscence, these patients should be treated with antibiotics.^4^ In this case the patient was immediately taken to the operating room and recovered without complication.

## Figures and Tables

**Figure:**
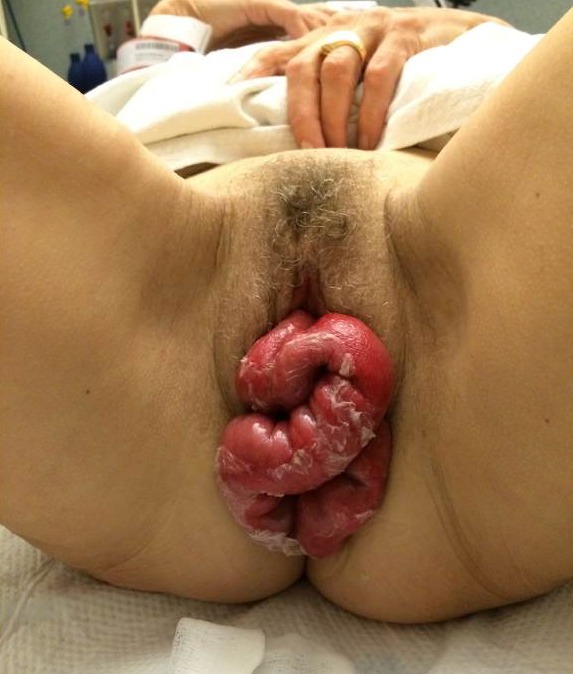
Intestinal tissue erythematous, edematous, non-necrotic and visibly peristalsing on exam.
